# Comparative evaluation of normal tissue objective functions in robotic radiosurgery planning for solitary brain tumors

**DOI:** 10.1002/acm2.70318

**Published:** 2025-10-24

**Authors:** Nadine Sonntag, Markus Eichner, Michael Judge, Martin Kocher, Daniel Ruess, Maximilian I Ruge, Stefan Hunsche

**Affiliations:** ^1^ Department of Stereotactic and Functional Neurosurgery University Hospital Cologne Cologne Germany; ^2^ Department of Radiation Oncology University Hospital Cologne Cologne Germany

**Keywords:** Cyberknife, non‐isocentric irradiation, optimization, stereotactic radiosurgery

## Abstract

**Background:**

The normal tissue objective (NTO) is an inverse planning approach in radiosurgery, also available for the CyberKnife system. By employing a model function, it aims to achieve precise control over the global dose fall‐off in healthy tissue. As a novel technique, NTO can serve as an alternative to the established method, which utilizes layered contours around the target to shape dose gradients and enhance conformity, referred to as Auto‐shells in CyberKnife systems.

**Purpose:**

This study compares the dose distribution achieved with NTO and Auto‐shells to evaluate their respective advantages in CyberKnife treatment planning.

**Methods:**

A total of 45 patients with brain tumors—including 15 vestibular schwannomas, 15 meningiomas, and 15 metastases, all of whom had previously been treated using an Auto‐shells‐generated plan, were analyzed. For each case, an alternative NTO‐based plan was generated and compared with its Auto‐shells counterpart. Key treatment parameters—including nodes, beams, total monitor units (MU), treatment time, new conformity index (nCI), gradient index (GI), and dose exposure volumes to healthy brain tissue (V12Gy and V5Gy)—were evaluated.

**Results:**

Both methods resulted in comparable plans across many indices. Significant differences were particularly in terms of healthy brain tissue dose exposure. With the NTO method, V12Gy and V5Gy were reduced by up to 14%, and in the case of meningiomas and metastases, the GI was reduced by up to 7%. The conformity, described by the nCI, was within 2%. No significant difference was observed in MU.

**Conclusion:**

NTO optimization presents a viable option to the Auto‐shells method for CyberKnife treatment of brain tumors. By reducing healthy brain tissue exposure without increasing monitor units, it enhances dose‐sparing efficiency. However, maintaining optimal conformity remains an important issue, highlighting the trade‐offs between precision and tissue preservation.

## 1 INTRODUCTION

In cranial radiosurgery, the precise delivery of radiation to the target volume while sparing the surrounding healthy brain tissue is important. Critical structures are located close to the target volume, and radiation exposure to healthy tissue can lead to radionecrosis.^1^, ^2^, ^3^, ^4^, ^5^, ^6^ The CyberKnife system is a robotic radiosurgery device that delivers highly precise radiation therapy for treating tumors, as well as certain non‐cancerous conditions, such as arteriovenous malformations and functional disorders.^7^, ^8^, ^9^, ^10^ To manage dose distribution in CyberKnife planning, the concept of “Auto‐shells” is commonly used.^11^, ^12^ These artificially defined, asymmetric or equidistant structures around the target volume act as virtual dose barriers, which are manually constructed and adapted to achieve the desired dose fall‐off during treatment planning. For each Auto‐shells plan, the number of shells must be specified, and for each shell, parameters such as the concentric distance from the target volume and the corresponding dose constraints must be individually defined. Achieving optimal dose constraints requires an intuitive understanding of dose gradients, which can be challenging. Altering one shell often necessitates adjustments to all other shells, resulting in a time‐consuming and complex, iterative planning process.

The “normal tissue objective” (NTO) planning mode offers an alternative approach. Instead of manually creating and adapting multiple shells, NTO uses a mathematical model to achieve an exponential dose fall‐off, enabling targeted dose control in normal brain tissue. Apart from the Cyberknife, NTO has also been introduced in the Eclipse planning software by Varian (Varian Medical Systems, Palo Alto, CA, USA), where its time‐saving benefits in treatment planning have already discussed in the literature.^13^, ^14^, ^15^, ^16^, ^17^, ^18^

This study investigates the application of NTO in CyberKnife planning, an area, to the best of our knowledge, currently lacking any published literature. We compare the quality of plans generated using NTO alone with those of clinically implemented Auto‐shell–based plans for brain tumors (meningiomas, metastases, and vestibular schwannomas). Our aim is to evaluate whether NTO provides dose distributions comparable to—or better than—current practice in terms of conformity, dose gradient, and normal‐tissue sparing, quantified by the volumes of healthy brain receiving ≥ 12 and ≥ 5 Gy (V12Gy and V5Gy, respectively), and thus may serve as an alternative for CyberKnife treatment planning.

## 2 MATERIALS AND METHODS

### 2.1 Patient selection

This study included 45 patients diagnosed with brain tumors who underwent CyberKnife radiosurgery between February 2023 and May 2024. The cohort comprised three distinct tumor types: 15 brain metastases (median tumor volume [TV]: 1.31 mL; range: 0.07–12.76 mL), 15 vestibular schwannomas (median TV: 1.63 mL; range: 0.23–4.31 mL), and 15 meningiomas (median TV: 6.07 mL; range: 3.34–11.79 mL). Each patient case has only one contiguous target structure. When selecting cases within the specified timeframe, care was taken to ensure that the tumors have a representative range of sizes and shapes commonly encountered in clinical practice.

### 2.2 Treatment planning overview

A clinically applied treatment plan was already available for each tumor, which was created with Precision software (version 3.3.1.3[2]) and CyberKnife VSI system (Accuray Inc., Sunnyvale, CA, USA) using inverse planning, Auto‐shells and VOLO algorithm for optimization.^11^ Treatment plans exclusively utilized fixed circular collimators, selected from 12 available collimator sizes, with field diameters ranging from 5 to 60 mm. Dose calculation was performed using a ray‐tracing algorithm.

For brain metastases, the prescribed dose was 20 Gy to the tumor margin, corresponding to an isodose level of 65% ± 2%. In contrast, meningiomas and vestibular schwannomas were treated with a prescribed dose of 13 Gy, delivered at an 80% ± 2% isodose level, aiming for 99.5% coverage of the planning target volume (PTV).

### 2.3 Auto‐shells supported optimization (clinically applied plan)

Typically, five Auto‐Shells (concentric ring structures) were employed as critical optimization structures, positioned at distances of 2 mm, 5 mm, 10 mm, 15 mm, and 25 mm from the tumor margin. Shells can also be planned asymmetrically; however, because this is used only rarely in our practice, we included only plans with symmetric shells in order to obtain a sufficiently large, analysable dataset. The planning process followed an iterative refinement strategy, in which the dose constraints for the Auto‐Shells were initially set loosely and progressively tightened throughout the optimization. The 2 mm shell was primarily used to optimize dose conformity, the 5‐ and 10‐mm shells to steepen the dose fall‐off in normal tissue, and the two outer shells (15 and 25 mm) to control dose spillage. The maximum number of beams was set to 300, and the maximum number of nodes to 133. The weights for the target and shells were initially set to 1.0. Target weights were then increased as needed, for example when coverage issues arose. Additionally, the MU penalty was initially set to 1.0 and reduced as needed to achieve better conformity by promoting the use of smaller collimators.

This stepwise approach was continued until the prescribed dose and target coverage were achieved, and no further improvements in dose gradient or conformity were attainable—indicative of a Pareto‐optimal solution. To mitigate undesired dose accumulation in peripheral regions, additional volumes of interest (VOIs) were defined and incorporated into the planning process as critical structures with specific dose constraints.

### 2.4 NTO supported optimization (retrospective plan)

For each tumor with a clinically applied Auto‐shells–based plan, a corresponding NTO‐based plan was generated for comparative analysis. For optimization also VOLO was used. Unlike the Auto‐Shells approach, which employs predefined shell structures, the NTO‐based plans relied solely on the predefined exponential dose fall‐off. It is possible to use both Auto‐shells and NTO simultaneously for optimization, but our comparative NTO‐plans did not have Auto‐shells to work out the potential of NTO. To ensure a direct comparison, the TV coverage, collimator settings, maximum monitor units (MU) per beam, and the maximum number of beams remained identical across both planning strategies. In addition, a new beam set was generated to avoid bias from the Auto‐Shells plan. In contrast to other planning software such as Eclipse from Varian, Precision does not provide automatic NTO optimization. Instead, the NTO dose fall‐off was manually defined using three reference points at varying distances from the tumor margin, each associated with a specific dose fall‐off percentage. Additionally, a weight parameter was introduced to integrate the dose fall‐off term into the optimization process, with the skin designated as the optimization VOI.

As previous studies on NTO optimization—particularly in the context of Eclipse (Varian)—have not identified universally optimal parameters,^13^, ^14^, ^15^, ^16^, ^17^ we did not attempt to establish an ideal parameter set. Instead, a structured iterative adjustment framework was employed, allowing users to rapidly become familiar with the NTO configuration and to systematically reproduce the optimization strategy. A typical initial NTO setup followed this scheme with the following necessary reference points: 100% at the tumor margin, decreasing to 50% at 5 mm, and 20% at 100 mm from the margin. Here, the 100% value corresponds to the prescription dose intended to conformally cover the target. The initial weight was set to 1.0. This baseline configuration generally resulted in a minor overshoot of the prescription isodose level under the constraint of ≥ 99.5% target coverage. To improve dose shaping, the fall‐off coefficient was gradually increased by lowering the isodose percentage at the second reference point, leading to an effective dose shift within the target and an elevation of the intratumoral maximum dose until the prescription isodose level was met. If the high‐dose region extended into surrounding normal tissue, the isodose value at the first reference point was reduced to apply a stricter constraint at the tumor margin. Concurrently, peripheral dose constraints (second and third reference points) were slightly relaxed, and individual weight adjustments were introduced to further fine‐tune the dose distribution. This iterative adjustment of the three reference points was continued until no further improvement could be achieved.

### 2.5 Plan comparison (NTO vs. auto‐shells)

The statistical analysis assessed significant differences between the treatment plans across multiple dosimetric and planning parameters, including the conformity index (CI), new conformity index (nCI), gradient index (GI), planning time, MU, and the volumes of healthy brain tissue receiving ≥ 12 Gy (V12Gy) and ≥ 5 Gy (V5Gy). We selected V12Gy as a widely reported surrogate for radiation‐induced brain‐injury risk, and V5Gy to characterize low‐dose spillage. The CI = PIV/TIV was calculated as the ratio of the prescribed isodose volume (PIV) to the TV covered by the prescribed isodose (TIV).^19^ The nCI was determined using the formula nCI = (PIV × TV)/(TIV^2^), where TV represents the target volume.^20^, ^21^ The GI was defined as V_pres/2_/V_pres_, where V_pres_ corresponds to the volume of the prescription isodose.^22^

For vestibular schwannomas, the mean dose to the cochlea was also evaluated, as cochlear sparing was actively optimized in all cases. In contrast, specific critical structures were not included in the analysis of meningiomas and metastases, given that not all tumors had adjacent critical structures. However, in cases where critical structures were present, the same optimization constraints were applied to maintain consistency across plans.

To determine the appropriate statistical approach, the Shapiro–Wilk test (*α* = 0.05) was performed to assess normality within the dataset. For normally distributed data, a paired *t*‐test (*α* = 0.05) was conducted to evaluate significant differences. If normality was not met, the Wilcoxon signed‐rank test for paired samples (*α* = 0.05) was applied as a non‐parametric alternative. All statistical analysis were conducted using Python 3.9.13.

The analysis was further stratified by tumor type—meningiomas, vestibular schwannomas, and metastases—to allow for a more detailed evaluation of treatment plan performance. This stratification accounted for potential variations in tumor characteristics and ensured a more nuanced interpretation of the results across distinct clinical scenarios. By analyzing each tumor type separately, this study aimed to provide a comprehensive understanding of how the different planning approaches performed in varying clinical contexts.

## 3 RESULTS

**FIGURE 1:**
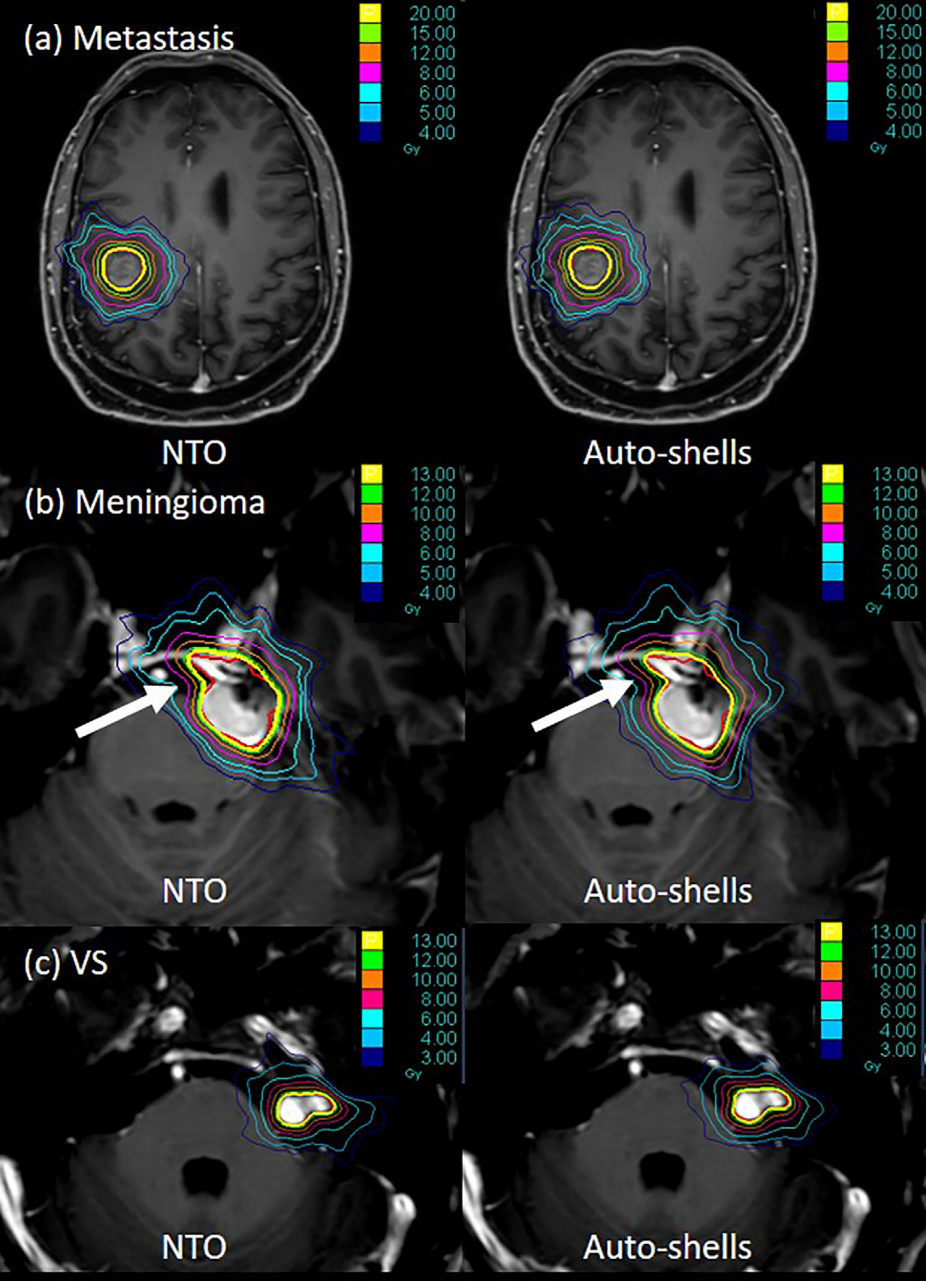
The figure shows the isodose distribution for a representative case from each of the three groups—(a) metastasis, (b) meningiomas, and (c) vestibular schwannomas—arranged row‐wise. Columns indicate the applied optimization algorithm, with normal tissue objective (NTO)‐based plans on the left and Auto‐shells‐based plans on the right. The arrow highlights a region of pronounced concavity in the target contour.

**FIGURE 2:**
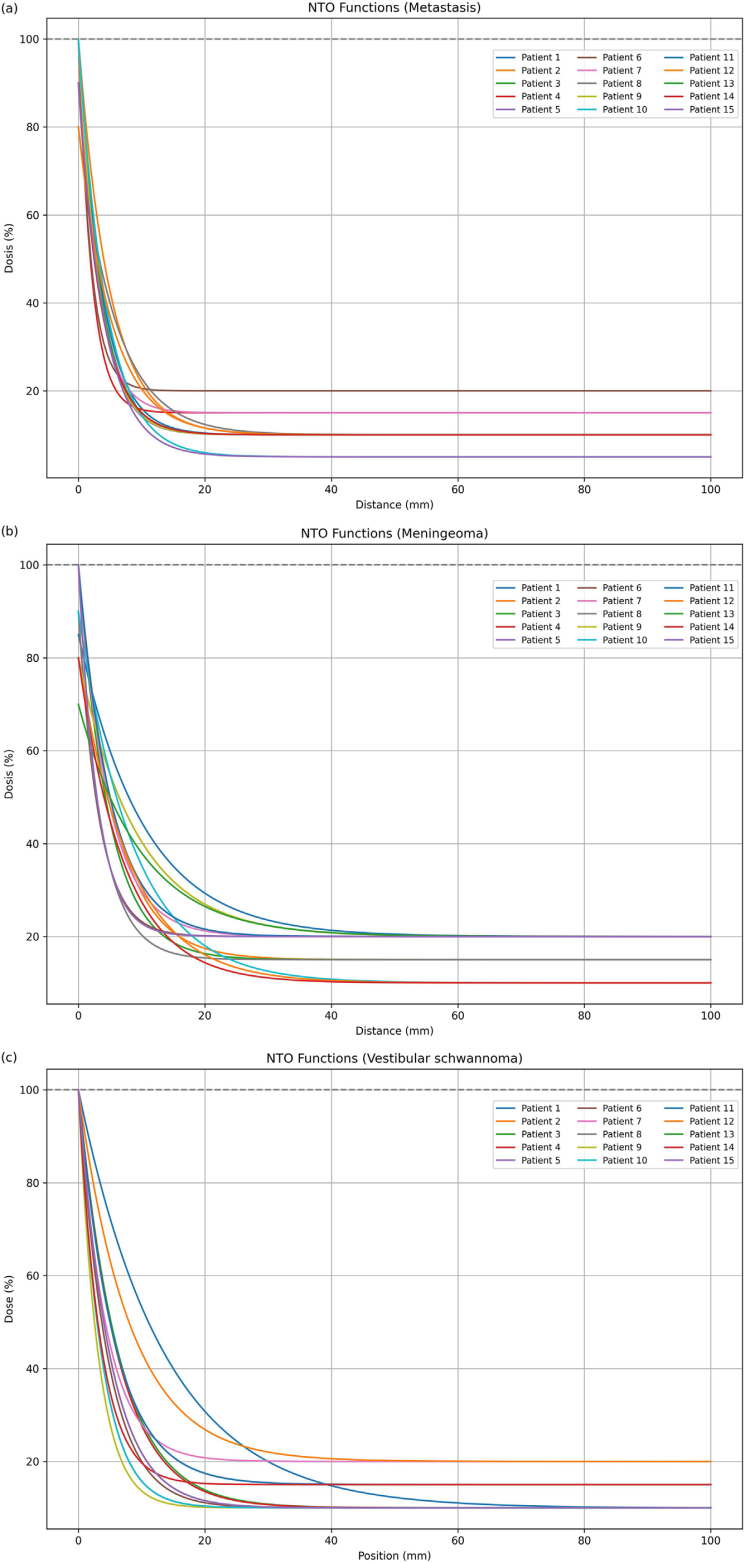
The figure shows the applied normal tissue objective (NTO) functions for all patients. (a) metastasis, (b) meningiomas, and (c) vestibular schwannomas.

Figure 1 illustrates exemplary optimization results using both NTO and Auto‐Shells, presenting one representative case from each of the three tumor types: metastasis, meningioma, and vestibular schwannoma. A preliminary visual comparison suggests that the isodose lines at different values in the Auto‐Shell plans tend to conform more closely to the tumor shape than those in the corresponding NTO plans—particularly in the metastasis and meningioma cases (white arrow), as shown in Figure 1. For vestibular schwannomas, the observed differences between the two planning approaches were minimal, which is also reflected in the respective example in Figure 1. The applied NTO functions for all patients are shown in Figure 2. A quantitative overview of the dose distribution is provided in Tables 1, 2, 3 to facilitate comparison of relevant planning indices and dosimetric metrics. A consistent trend can be observed when examining the indices that quantify dose exposure to healthy brain tissue: NTO optimization achieved a significant reduction in both V12Gy and V5Gy, regardless of tumor type. For meningiomas, the reduction in V12Gy exceeded 11%, while for vestibular schwannomas, it reached over 14%. In metastases, dose exposure was reduced by > 5% in both V12Gy and V5Gy. Additionally, a significant improvement in the GI were observed for meningiomas (> 7% reduction) and metastasis (> 4% reduction). Despite an observed improvement of over 2% in GI for vestibular schwannomas, the change was not statistically significant.

**TABLE 1:** Mean and standard deviation values of plan parameters for meningiomas, optimized using Auto‐shells, and normal tissue objective (NTO).

Parameter	Auto‐shells	NTO	*p*‐value
CI	1.26 ± 0.11	1.24 ± 0.10	*p* < 0.01
nCI	1.26 ± 0.10	1.24 ± 0.10	*p* < 0.01
GI	3.44 ± 0.59	3.19 ± 0.43	*p* < 0.001
D_min_ (Gy)	12.43 ± 0.24	12.51 ± 0.27	n.s.
V12Gy (cm^3^)	2.25 ± 1.09	1.99 ± 0.99	*p* < 0.001
V5Gy (cm^3^)	17.20 ± 5.70	15.25 ± 4.62	*p* < 0.001
Monitor units	15762 ± 3664	15850 ± 4042	n.s.
Treatment time (min)	54.13 ± 6.44	48.80 ± 8.04	*p* < 0.001
Number of nodes	93 ± 8	82 ± 11	*p* < 0.001
Number of beams	211 ± 29	162 ± 32	*p* < 0.001

Abbreviations: CI, conformity index; GI, gradient index; nCI, new conformity index.

**TABLE 2:** Mean and standard deviation values of plan parameters for vestibular schwannomas, optimized using Auto‐shells, and normal tissue objective (NTO).

Parameter	Auto‐shells	NTO	*p*‐value
CI	1.22 ± 0.08	1.22 ± 0.07	n.s.
Nci	1.22 ± 0.08	1.22 ± 0.07	n.s.
GI	4.33 ± 0.71	4.23 ± 0.73	n.s.
D_min_ (Gy)	12.53 ± 0.23	12.51 ± 0.48	n.s.
V12Gy (cm^3^)	0.35 ± 0.40	0.30 ± 0.32	*p* < 0.01
V5Gy (cm^3^)	4.76 ± 4.52	4.23 ± 3.77	*p* < 0.05
Monitor units	12622 ± 2076	12655 ± 2508	n.s.
Treatment time (min)	41.20 ± 6.17	39.80 ± 6.87	n.s.
Number of nodes	67 ± 10	63 ± 8	*p* < 0.05
Number of beams	147 ± 29	132 ± 30	*p* < 0.01
Cochlea D_mean_ (Gy)	4.37 ± 0.52	4.37 ± 0.56	n.s.

Abbreviations: CI, conformity index; GI, gradient index; nCI, new conformity index.

**TABLE 3:** Mean and stasndard deviation values of plan parameters for metastasis, optimized using Auto‐shells, and normal tissue objective (NTO).

Parameter	Auto‐shells	NTO	*p*‐value
CI	1.19 ± 0.09	1.18 ± 0.09	n.s.
Nci	1.19 ± 0.10	1.18 ± 0.09	n.s.
GI	3.23 ± 0.73	3.10 ± 0.70	*p* < 0.1
D_min_ (Gy)	19.79 ± 0.15	19.77 ± 0.21	n.s.
V12Gy (cm^3^)	2.39 ± 3.42	2.26 ± 3.15	*p* < 0.01
V5Gy (cm^3^)	10.43 ± 13.60	9.85 ± 12.91	*p* < 0.05
Monitor units	12059 ± 4937	11567 ± 4516	n.s.
Treatment time (min)	38.73 ± 11.25	36.13 ± 11.07	*p* < 0.05
Number of nodes	72 ± 15	64 ± 17	*p* < 0.01
Number of beams	136 ± 55	118 ± 49	*p* < 0.05

Abbreviations: CI, conformity index; GI, gradient index; nCI, new conformity index.

Regarding conformity, the results varied. A small but significant improvement in nCI of ≈ 1.6% was observed only for meningiomas with NTO optimization. No significant difference was found for metastasis and vestibular schwannomas. Notably, in a few cases, NTO struggled to generate conformal plans—particularly in tumors with highly concave geometries. One such example is illustrated in Figure 1, middle row, a meningioma case, where regions with pronounced concavities failed to receive sufficient dose conformity using NTO (white arrow). In these instances, the Auto‐shell optimization enabled more precise dose shaping around irregular tumor margins, ensuring better adaptation of the prescribed isodose to the target boundaries. However, it should be noted that NTO optimization resulted in a steeper dose gradient outside the target in this case, indicating improved dose fall‐off control in the surrounding tissues.

A reduction in treatment time was also observed with NTO optimization. This reduction was statistically significant for meningiomas, with a 10% decrease, and for metastases, with a 7% decrease. In vestibular schwannomas, a modest 3% reduction was also observed, although this difference did not reach statistical significance. The number of nodes and beams used per plan was also significantly reduced with NTO optimization. In contrast, the total number of MU did not differ significantly between the two planning approaches.

## 4 DISCUSSION

We assessed the impact of replacing Auto‐shells optimization with NTO on plan quality and treatment efficiency in a cohort of 45 patients with meningiomas, vestibular schwannomas, and metastases, each defined by a single‐target delineation. This study provides an introduction to NTO‐based planning using Precision and CyberKnife, and demonstrating that NTO‐based optimization can produce treatment plans of comparable overall quality. However, it also showed that significant differences exist in certain planning objectives, reflecting the fundamentally distinct approaches of the two optimization strategies. The difference lies in their handling of dose fall‐off beyond the target volume. NTO optimization employs a more global approach by defining a single dose fall‐off function that applies uniformly across a defined region outside the tumor, steering dose distribution in a generalized manner. In contrast, the Auto‐shells use a more localized strategy by defining in our study concentric structures around the tumor, allowing for a more detailed and spatially controlled modulation of the dose gradient at specified distances. Our findings suggest that NTO may be a viable option is when the primary goal is the sparing of healthy brain tissue on a global scale. This is supported by significantly lower values in normal brain tissue exposure, as reflected in V12Gy and V5Gy metrics. The volume exposure is relatively low, and it is difficult to derive clear clinical significance from it. However, Blonigen et al. and Minniti et al.^23^, ^24^ have demonstrated that the risk of radionecrosis increases gradually with increasing volumes of tissue receiving 10 Gy (V10) and 12 Gy (V12). In this context, even small reductions in V12Gy may be considered beneficial. Importantly, the additional significant reduction in V5Gy—used as a marker of low‐dose spillage—may be clinically relevant, particularly for benign tumors.^25^ Furthermore, for meningiomas and metastases, NTO optimization resulted in a significantly reduced GI. However, achieving a favorable nCI while maintaining a good GI and healthy brain tissue sparing proved to be more challenging with only NTO optimization. In this context, Auto‐shells appear to be more appropriate. They enable highly conformal planning even for strongly concave tumor regions, as illustrated in the middle row of Figure 1. A more localized dose modulation, achievable by placing a shell in close proximity to the tumor margin—as in the Auto‐shell method—yield better results in such case. These findings partially contradict previous reports in the literature. For example, Caldeira et al.^18^ observed no significant changes when using NTO; but, their study focused on prostate target volumes, which differ substantially from intracranial indications. In contrast, Indrayani et al.^15^ reported a significantly improved normal tissue sparing with a steeper dose gradient when using NTO. However, they did not observe improved conformity when applying manual NTO. Other studies did not compare NTO with ring structures directly but rather evaluated different planning techniques using manual and automatic NTO using the Eclipse planning system.^13^, ^14^, ^16^, ^17^ Comparing results between NTO implementations in Eclipse and Precision is inherently difficult due to several factors: the underlying algorithm is not publicly disclosed, the optimization engines differ, user input parameters vary, and the radiation delivery techniques are fundamentally different. Accordingly, the findings from the existing literature are not readily transferable to CyberKnife and Precision, as also demonstrated by our results.

In our cohort, NTO optimization was associated with shorter treatment times and fewer nodes and beams. Based on the available data, we cannot ascribe a single definitive reason for this observation. A plausible explanation is that NTO's less localized objective function allows the optimizer to select a sparser, more efficient beam set that may align more closely with the system's preferred beam directions. By contrast, Auto‐Shells impose ring‐specific dose constraints at fixed offsets from the target. These more localized constraints can be harder to satisfy in regions with complex geometry and may prompt the optimizer to recruit additional beams and nodes and prolong delivery to sculpt the isodose surfaces more precisely. This interpretation is hypothesis‐generating and would require controlled, algorithm‐level analyses to confirm. Consistent with this interpretation, Figure 1 (meningioma and metastasis examples) shows that isodose lines in NTO‐based plans appear less smooth and less conformal to the tumor boundary than those in the Auto‐shell plans. Interesting, the total number of MU remained unchanged. This is consistent with the findings of Indrayani et al.,^15^ who observed that, in the case of brain tumors, the use of manual NTO did not require more MUs compared to planning with ring structures.

Nevertheless, no universally optimal parameters for NTO settings could be identified, as seen in the representation of the NTO functions in Figure 2. Possible reasons for this variability include differences in tumor size, location, and complexity, which likely played a decisive role in determining the optimization parameters that varied considerably from case to case.

### 4.1 Limitations

This study was limited to single‐target treatments; we did not evaluate NTO in multi‐target scenarios (e.g., multiple metastases). In addition, Auto‐Shell plans were produced under routine clinical conditions, whereas NTO plans were generated outside the clinical workflow for this analysis, which may affect comparability.

## 5 CONCLUSIONS

NTO optimization offers a viable option to the pure Auto‐shells method for CyberKnife treatment of single brain tumors, introducing a distinct approach to dose shaping. By leveraging its inherent assumptions about dose fall‐off, NTO enables a significant reduction in healthy brain tissue exposure—as measured by V12Gy and V5Gy—without increasing treatment time or monitor units. However, while effective in dose sparing, NTO falls short in some cases achieving optimal conformity. Particularly in cases with highly concave tumor margins.

## AUTHOR CONTRIBUTIONS

Nadine Sonntag and Stefan Hunsche: Conception, design, acquisition of data, statistical analysis, and interpretation of data. Drafting of the article and figures. Markus Eichner, Michael Judge, Martin Kocher, Daniel Ruess, and Maximilian I Ruge: Acquisition of data, revision of the manuscript, and final approval of the version to be submitted.

## CONFLICT OF INTEREST STATEMENT

Michael Judge reports being a former employee and current stockholder of Accuray.

## ETHICS STATEMENT

Study approval statement and consent to participate statement: The study is strictly retrospective. As per the Ethics Committee of the University of Cologne, there is no requirement to obtain ethics approval or informed consent from patients for this strictly retrospective study. The research was conducted in accordance with the Declaration of Helsinki and Good Clinical Practice guidelines.
